# Synaptic Plasticity in the Hippocampus of a APP/PS1 Mouse Model of Alzheimer's Disease Is Impaired in Old but Not Young Mice

**DOI:** 10.1371/journal.pone.0009764

**Published:** 2010-03-22

**Authors:** Simon Gengler, Alison Hamilton, Christian Hölscher

**Affiliations:** School of Biomed Sciences, Ulster University, Coleraine, United Kingdom; Sapienza University of Rome, Italy

## Abstract

**Background:**

Alzheimer disease (AD) is a neurodegenerative disorder for which there is no cure. We have investigated synaptic plasticity in area CA1 in a novel AD mouse model (APPPS1-21) which expresses the Swedish mutation of APP and the L166P mutation of human PS-1. This model shows initial plaque formation at 2 months in the neocortex and 4 months in the hippocampus and displays β−amyloid-associated pathologies and learning impairments.

**Methodology/Principal Findings:**

We tested long-term potentiation (LTP) and short term potentiation (paired-pulse facilitation, PPF) of synaptic transmission *in vivo* in area CA1 of the hippocampus. There was no difference in LTP or PPF at 4–5 months of age in APPPS1-21 mice compared to littermate controls. At 6 months of age there was also no difference in LTP but APPPS1-21 mice showed slightly increased PPF (p<0.03). In 8 months old mice, LTP was greatly impaired in APPPS-21 animals (p<0.0001) while PPF was not changed. At 15 months of age, APPPS1-21 mice showed again impaired LTP compared to littermate controls (p<0.005), and PPF was also significantly reduced at 80 ms (p<0.005) and 160 ms (p<0.01) interstimulus interval. Immunohistological analysis showed only modest amyloid deposition in the hippocampus at 4 and 6 months with a robust increase up to 15 months of age.

**Conclusions:**

Our results suggest that increased formation and aggregation of beta amyloid with aging is responsible for the impaired LTP with aging in this mouse model, while the transient increase of PPF at 6 months of age is caused by some other mechanism.

## Introduction

Alzheimer disease (AD) is a neurodegenerative disorder for which there is no cure. Two of the hallmarks are the accumulation of β-amyloid plaques and of tau tangles [1], [2]. APPPS1-21 mice are a recently generated transgenic mouse model on a C57BL/6J genetic background that co-expresses the KM670/671NL mutated human amyloid precursor protein and the L166P mutated human presenilin 1 (APPPS1-21 mice) and model aspects of cerebral amyloidosis. Plaque formation starts after 2 months in the neocortex and 4 months in the hippocampus, and the ratio of β-amyloid42 to β-amyloid40 is 1.5 in young and 5 in amyloid-depositing older mice, respectively. Consistent with this ratio, extensive congophilic parenchymal amyloid but minimal amyloid angiopathy is observed [3], [4]. Amyloid-associated pathologies include dystrophic synaptic boutons, hyperphosphorylated tau-positive neuritic structures and robust gliosis and microglia numbers. Global neocortical neuron loss is not apparent in this mouse model, but local neuronal loss in the dentate gyrus is observed at 17 months of age (Rupp et al., 2009). Furthermore, spatial memory deficits in a plus maze task is apparent at the age of 8 months [3]. These findings suggest that the overproduction of β-amyloid, the formation of plaques and the subsequent inflammation response is sufficient to produce a series of symptoms that are consistent with those observed in patients with AD.

In this study, we have investigated synaptic plasticity in APPPS1-21 mice. Synaptic plasticity is a form of rapid upregulation of neurotransmission after repetitive stimulation of axonal projections that is considered to be related to the cellular changes that underlie memory formation [5], [6]. The accumulation of plaques, the development of the inflammation response, and the impairment in memory formation would suggest that synaptic activity will be affected. However, studies of mouse models of AD that also overexpress human mutated APP show mixed results. A mouse model that expresses the KM670/671NL human mutated APP showed no impairment in LTP in area CA1 up to 24 months of age [7]. In contrast, a mouse model that expresses the Swedish APP K595N, M596L double mutation showed impaired LTP in the dentate gyrus and the area CA1 in 15–17 months old mice [8]. Another mouse model carrying the Swedish mutated human APP and the PS1 (A246E) transgenes showed that LTP *in vitro* in area CA1 was unaffected, and *in vivo* LTP the dentate gyrus was only slightly effected in 18 months old mice [9]. In a mouse model that expresses the Swedish APP and the PS1 delta E9 transgenes, *in vitro* LTP in area CA1 was hardly affected at all [10]. These findings underscore the differences between different transgenic constructs, and show that the formation of β-amyloid plaques is no guarantee that synaptic impairments develop in the brains of tg mice. We therefore set out to investigate if short-term potentiation (paired-pulse facilitation) or long-term potentiation of synaptic transmission in area CA1 *in vivo* is affected in the APPPS1-21 mice and correlated that with beta-amyloid plaque load in the hippocampus.

## Materials and Methods

### Animals

APPPS1-21 mice with a C57/Bl6 background were obtained from Prof. M. Jucker and bred at the Ulster University animal unit. Heterozygous males APPPS1 mice were bred with wild-type C57/Bl6 females bought locally (Harlan, UK). Offspring was tail snipped and genotyped using PCR with primers specific for the APP-sequence (Forward:“GAATTCCGACATGACTCAGG”, Reverse: “GTTCTGCTGCATCTTGGACA”). Positive heterozygous animals and negative wild type littermate controls were used for the experiments. After the end of the experiments, the PCR results were confirmed by histology using Congo-red stain (see below).

All experiments were licensed by the UK home office in according to the animal (scientific procedures) Act of 1986.

### Surgery and LTP recording in the hippocampus area CA1

Mice were anaesthetised with urethane (ethyl carbamate, 1.8 g/kg, *i.p.*) for the duration of all experiments. The skull was exposed and 3 holes with 0.8 mm diameter were drilled. Electrodes (tungsten with Teflon coating, Bilaney, Kent, UK) were implanted in the following coordinates: 1.5 mm posterior and 1.0 mm lateral to the midline for the recording electrode, and the stimulating electrode 2.0 mm posterior to bregma and 1.5 mm lateral to the midline. The earth electrode location was at 3.0 mm posterior to bregma and 2.5 mm lateral to the midline, contralateral to the electrode sites. The electrodes were slowly lowered through the cortex and the upper layers of the hippocampus and into the CA1 region (approx. 1.2 mm) until the appearance of a negative deflecting excitatory postsynaptic potential (EPSP) that had a latency of ca. 10 ms. Recordings of field excitatory postsynaptic potentials (fEPSPs) were made from the stratum radiatum in the CA1 region of the right hippocampal hemisphere in response to stimulation of the Schaffer collateral/commissural pathway. fEPSPs were recorded on a computerised stimulating and recording unit (PowerLab, ADI instruments) in which the trigger threshold was adjustable. The triggered unit activated a constant current stimulus isolation unit (Neurolog, UK). The data acquisition system was triggered simultaneously to record all events. Sampling speed was at 20 kHz for recordings of fEPSPs. The high frequency stimulation protocol for inducing LTP consisted of 1 train of 100 stimuli, inter-stimulus interval 5 ms (200 Hz). This rather weak LTP induction protocol was chosen to prevent saturation of the LTP and thus allow the possibility to detect improvements as well as impairments of the LTP in the APPPS1-21 mice. Stimulation intensity was 60% of the max. fEPSP, as analysed by establishing an input-output correlation. LTP was measured as % of baseline fEPSP slope recorded over a 15 min period prior to application of high frequency stimulation. This value was taken as 100% of the EPSP slope and all recorded values were normalised to this baseline value. Basic synaptic transmission was tested by stimulating with a range of intensities.

Paired-pulse facilitation (PPF) was measured to analyse pre-synaptic functions and interneuron activity. Two stimuli were given at 60% of max fEPSP response. The interval between two stimuli was changed from 25 ms to 50, 80, 120, 160 and 200 ms to analyse PPF in relation to time. The PPF induced at short interstimulus intervals is considered to be triggered by pre-synaptic transmitter release facilitating processes [11], while later PPF is considered to be linked to GABAa and GABAb interneuronal synaptic transmission [12], [13]. The size of the fEPSP response was measured by analysing the change from baseline to the lowest point of the fEPSP. Data were normalised by taking the first fEPSP value as 100% and comparing the second fEPSP with it.

### Histology

After the LTP studies, animals were perfused transcardially with PBS buffer followed by ice-cold 4% paraformaldehyde in PBS. Brains were removed and fixed in 4% paraformaldehyde in PBS. Brains were left in a 30% sucrose solution until they had sunk to the bottom of the container. Brains were shock frozen and cut at 40 µm on a Leica cryostat. For stereological analysis, the first section was taken at random and every 5^th^ section taken afterwards. Sections were analysed using the optical fractionator technique with twodimensional disectors (microglia), as described previously [14], [15]. Sections were stained for plaques by a rabbit anti-Amyloid beta-Peptide polyclonal antibody (1∶250, Invitrogen, UK, 71-5800), using the protocols published in [3].

Prior to staining, slices were incubated in 99% formic acid for 7 minutes for antigen retrieval; this procedure is known to enhance antibody recognition of Amyloid beta [16]. For the stereological analysis, photographs of the hippocampus of each section were taken on an Olympus CX 40 microscope using the 10x magnification lens ( = 25 x magnification) and a computer controlled digital 5.1 MegaPix camera. Averages of all section values were taken as the average value for each mouse brain. For better demonstration of the anatomy of the hippocampus, a standard background Hematoxylin and Eosin staining [17] was performed in some sections.

### Statistics

All data were analysed using the program Prism (Graphpad software Inc., USA).

LTP data were analyzed with 2-way repeated measure mixed model ANOVA with genotype as the between subject factor and time as the within-subject factor. The LTP data were analyzed for the whole 60 minutes recorded after HFS, as well as for the time sections of 1–30, 31–60, 1–20, 21–40 and 41–60 minutes after HFS. PPF data of APPPS1-21 and control littermates were analysed using a 1-way ANOVA and a post hoc Yuen's test [18], [19] for every interstimulus interval. Data were log-transformed if the data variation of the tg and wt groups was different between groups.

## Results

### Basic synaptic transmission - input output correlations

**Figure 1:**
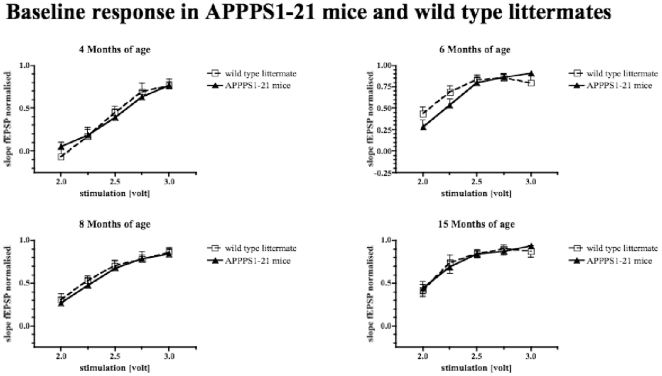
Input-output correlation in different age groups of APPPS1-21 mice and their wild type littermate controls. There was no difference found in baseline transmission at any age group (2-way repeated measure ANOVA, p>0.05).

The basic synaptic response of different age groups of APPPS1-21 and their wild type littermates were compared. At no age group there was a difference between groups in two-way repeated-measure ANOVAs. In all groups, fEPSPs increased in correlation with stimulus increase. 4.5 months age group: Genotype: F_1,56_ = 0.004; p>0.05; stimulus intensity: F_4,56_ = 59.69; p<0.0001. 6 months: Genotype: F_1,64_ = 0.057; p>0.05; stimulus intensity: F_4,64_ = 31.16; p<0.0001. 8 months: Genotype: F_1,64_ = 0.11; p>0.05; stimulus intensity: F_4,64_ = 66.3; p<0.0001. 15 months age group: Genotype: F_1,56_ = 0.003; p>0.05; stimulus intensity: F_4,56_ = 24.15; p<0.0001 (see fig. 1).

### LTP in 4.5 months old animals

**Figure 2:**
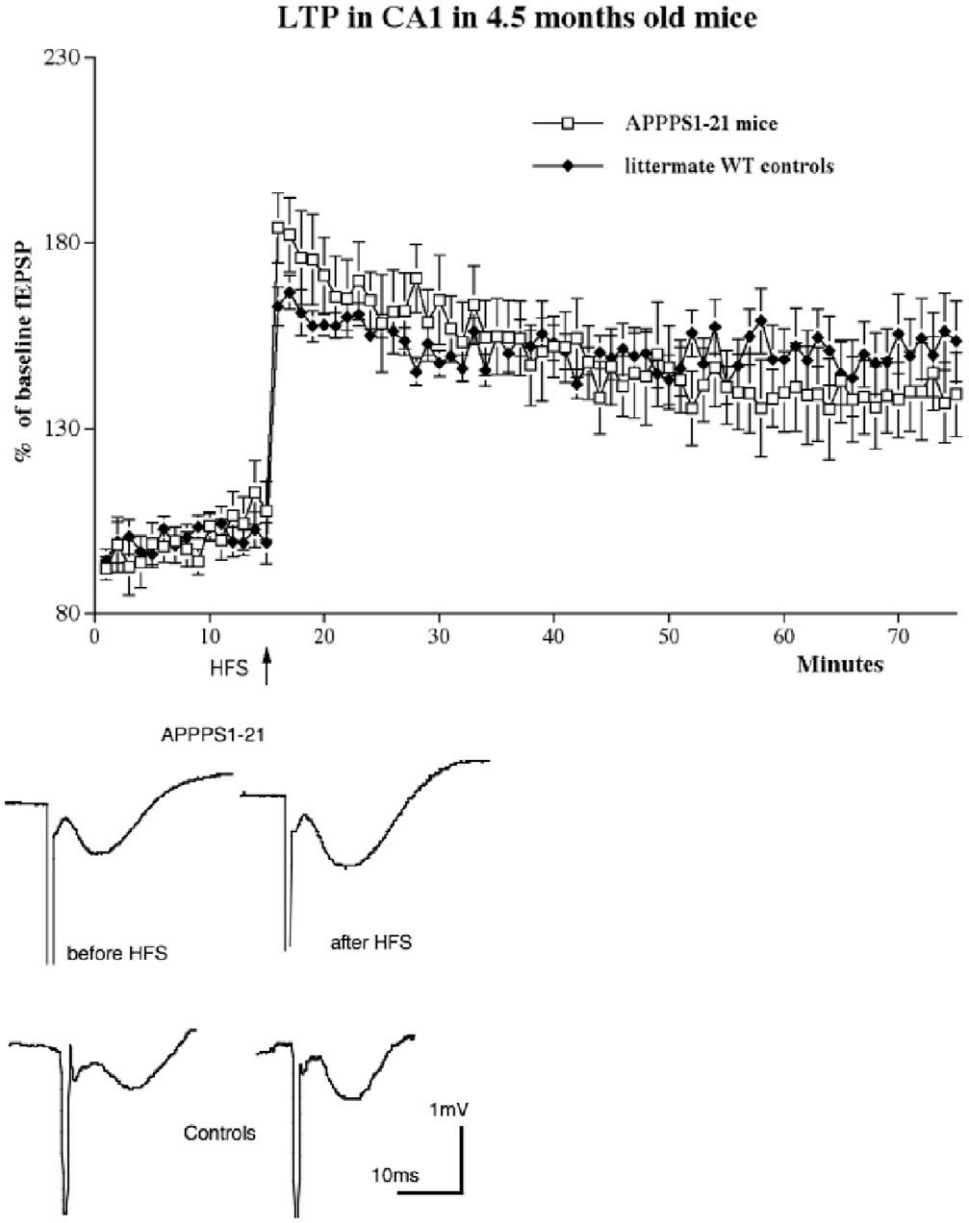
Inducing LTP in area CA1 in APPPS1-21 mice or littermate wild type controls aged 4.5 months showed robust LTP that lasted for 1 h post-HFS (n = 8 per group). A 2-way repeated measures ANOVA did not show a difference between genotypes but over time (p<0.0001). Below: Shown are typical field fEPSPs of recordings 5 min before (1) and 1 h after HFS (2).

**Figure 3:**
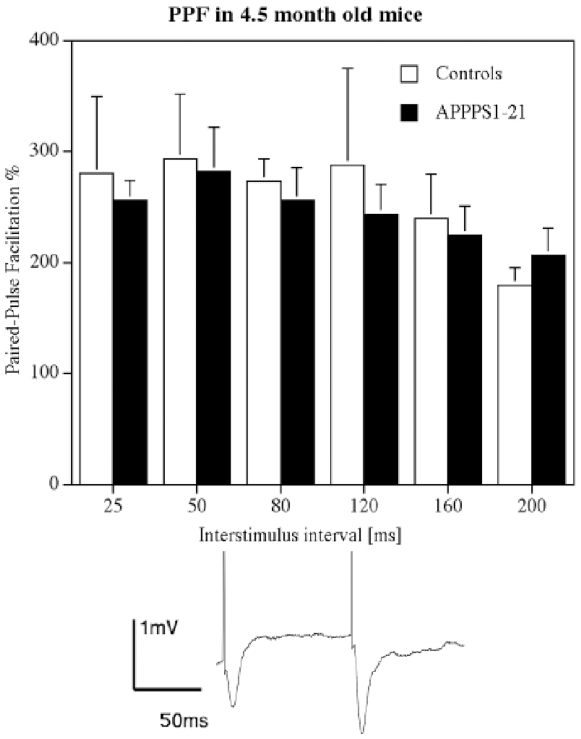
Paired-pulse facilitation in area CA1 in 4.5 moths old animals. The interstimulus interval was set at 25–200 ms. Analysis by 1-way ANOVA did not find an overall difference. A sample trace is shown which demonstrates paired-pulse facilitation at 80 ms interstimulus interval.

In 4–5 months old animals, HFS stimulation of the Schaffer collaterals induced robust LTP in both groups. A 2-way repeated measures mixed model ANOVA did not find a difference between groups (genotype) but over time (F_119,1309_  = 1.849, P<0.0001), showing a decay in LTP in both groups. The interaction between the factors was not significant (n = 8 per group) (fig. 2). The paired-pulse facilitation study showed good paired-pulse facilitation at all interstimulus intervals tested (25, 50, 80, 120, 160, 200 ms). A 1-way ANOVA did not find any overall difference, and neither Yuen's post hoc test showed differences between genotypes for PPF at any interstimulus interval (see fig. 3).

### 6 months old animals

**Figure 4:**
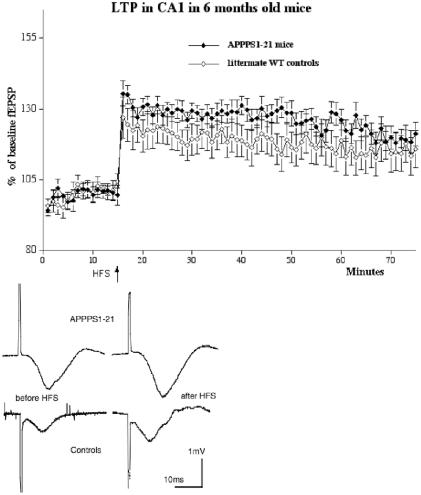
Inducing LTP in area CA1 in APPPS1-21 mice or littermate wild type controls aged 6 months showed robust LTP (n = 9 per group). A 2-way repeated measures ANOVA did not find a difference between genotypes. Below: Shown are typical field fEPSPs of recordings 5 min before (left) and 1 h after HFS (right).

**Figure 5:**
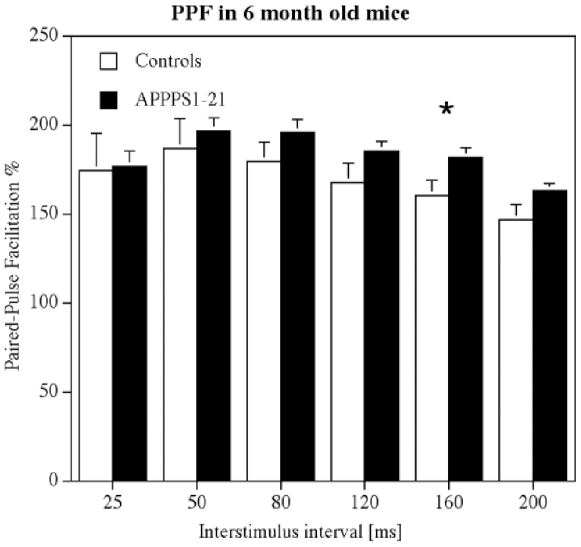
Paired-pulse facilitation in area CA1 in 6 months old animals. The interstimulus interval was set at 25–200 ms. A 1-way repeated measures ANOVA showed a difference between groups (p<0.01). The 160 ms interstimulus interval showed a difference between groups (p<0.03) in a post-hoc Yuen's test, demonstrating increased PPF in the APPPS1-21 group.

HFS induced good LTP in both groups (n = 9 per group). There was a trend for the APPPS1-21 group to show stronger LTP. A 2-way repeated measures ANOVA did not find a difference between genotypes nor over time, but the interaction between the two factors was significant in the LTP consolidation phase (minutes 40–60 post-HFS; F_39,429_ = 1.54, p<0.02), showing that the effect that time had on the development of LTP was dependent on the genotype (see fig. 4). The paired-pulse facilitation study showed good facilitation at all interstimulus intervals tested (25, 50, 80, 120, 160, 200 ms). A 1-way repeated measures ANOVA showed a difference between groups (F_11,1309_ = 2.5, p<0.01). APPPS1-21 animals had stronger PPF than littermate controls at the 160 ms interstimulus interval, Yuen's post-hoc test showed a difference between groups (t_10_  = 2.53, p<0.03) (see fig. 5).

### 8 months old animals

**Figure 6:**
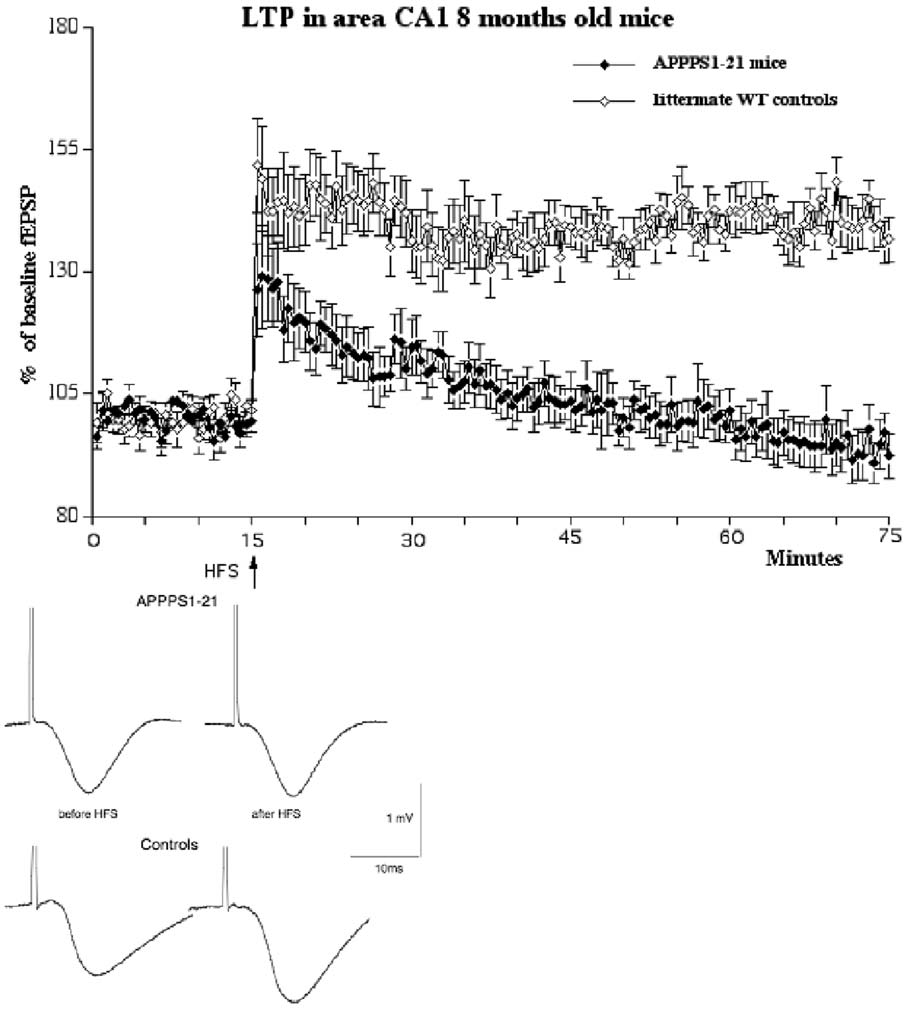
Inducing LTP in area CA1 in APPPS1-21 mice or littermate wild type controls aged 8 months showed robust LTP that lasted for 1 h post-HFS (n = 12 per group). A 2-way repeated measures ANOVA showed a difference between genotype (p<0.001) over time (p<0.0001), showing that there was a decay of LTP in both groups. Below: Shown are typical field fEPSPs of recordings 5 min before (left) and 1 h after HFS (right).

**Figure 7:**
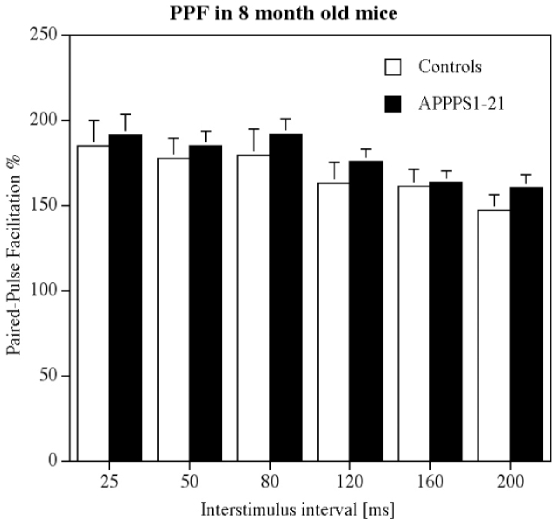
Paired-pulse facilitation in area CA1 in 8 months old animals. A 1-way repeated measures ANOVA showed a difference between groups (p<0.005), but post hoc tests showed no differences between genotypes at any interstimulus interval.

In 8 months old animals, HFS stimulation of the Schaffer collaterals induced robust LTP in both groups. A 2-way repeated measures mixed model ANOVA did not find a difference between groups (genotype) but found a difference over time (F_119, 1428_ = 4.9 p<0.0001), showing that there was a decay in LTP in both groups. The interaction between the factors was not significant (n = 13 per group) (fig. 6). The paired-pulse facilitation study showed good facilitation at all interstimulus intervals tested (25, 50, 80, 120, 160, 200 ms). A 1-way repeated measures ANOVA showed a difference between groups (F_11_ = 2.8, p = 0.003). However, a post hoc Yuen's tests did not show differences between genotypes for PPF at any interstimulus interval (see fig. 7).

### 15 months old animals

**Figure 8:**
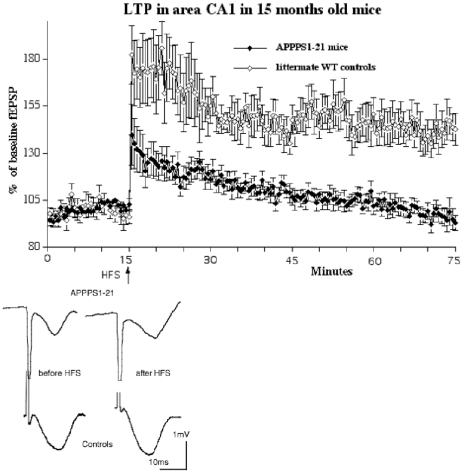
In 15 months old animals, HFS did not induce LTP in the APPPS1-21 animals (n = 8 per group). There was a difference between genotypes (p<0.01) and a difference over time (p<0.0001). There was no interaction between these factors.

In 15 months old animals, HFS did not induce LTP in the APPPS1-21 animals. APPPS1-21 mice showed no LTP at 60 min post-HFS (94+/−4% SEM of baseline), while controls still showed stable LTP (126+/−7%). A 2-way repeated measures mixed model ANOVA find a difference between genotypes after log transformation (F_119,833_ = 4.837, p<0.05) and a difference over time (F_119,823_ = 7.517, p<0.0001), showing that there was a decay in LTP in both groups. There was no interaction between the factors (n = 8 per group). When separating the 60 min post- HFS recordings into the LTP induction phase (1–20 min), a transition phase (21–40 min) and the consolidation phase (41–60 min), it was found in the induction phase that there was no difference between genotypes, but again a difference over time (F_39,273_ = 3.311, p<0.0001). In the transition phase (21–40 min) of LTP, there was a significant difference between groups (F_1,273_ = 5.4, p<0.05) and an interaction of genotype and time (F_39,273_ = 1.683, p = 0.007). A similar result was found in the consolidation phase (41–60 min) of LTP, there was a significant difference between groups (F_1,273_ = 4.74, p<0.05) and also over time (F_39,273_ = 2.25, p<0.0001), showing that APPPS1-21 animals had reduced LTP compared to controls. The interaction between the factors was not significant (fig. 8).

**Figure 9:**
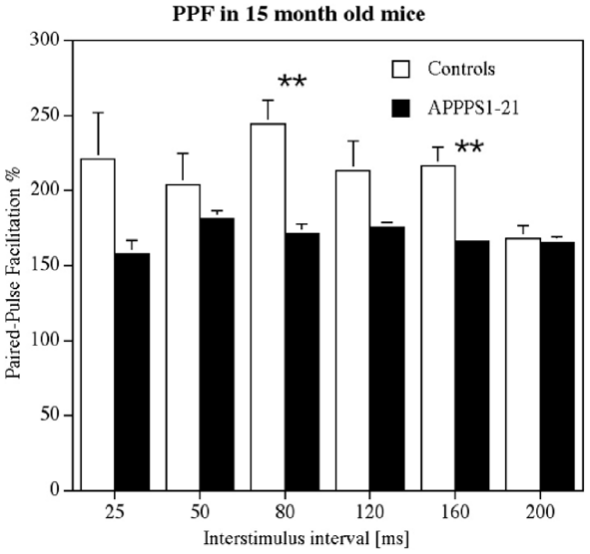
Paired-pulse facilitation in area CA1 in 15 months old animals. APPPS1-21 mice were severely impaired in PPF. A 1-way repeated measures ANOVA showed a difference between groups (p<0.05). Yuen's post-hoc tests showed highly significant differences between genotypes at 80 ms (p<0.01) and 160 ms (p<0.01) interstimulus interval.

A 1-way repeated measures ANOVA showed a difference between groups (F_11_ = 2.3, p<0.05). APPPS1-21 animals showed impaired PPF compared with littermate controls at 80 ms and 160 ms interstimulus interval as shown in post-hoc tests: At the interstimulus interval 80 ms, Yuen's test (t_6.17_ = 3.696, p<0.01) showed a highly significant difference between genotypes, and at the interstimulus interval 160 ms Yuen's test (t_5.65_ = 3.02, p<0.03) discovered a significant impairment of PPF in the APPPS1-21 mice as well (fig. 9).

### Histology

**Figure 10:**
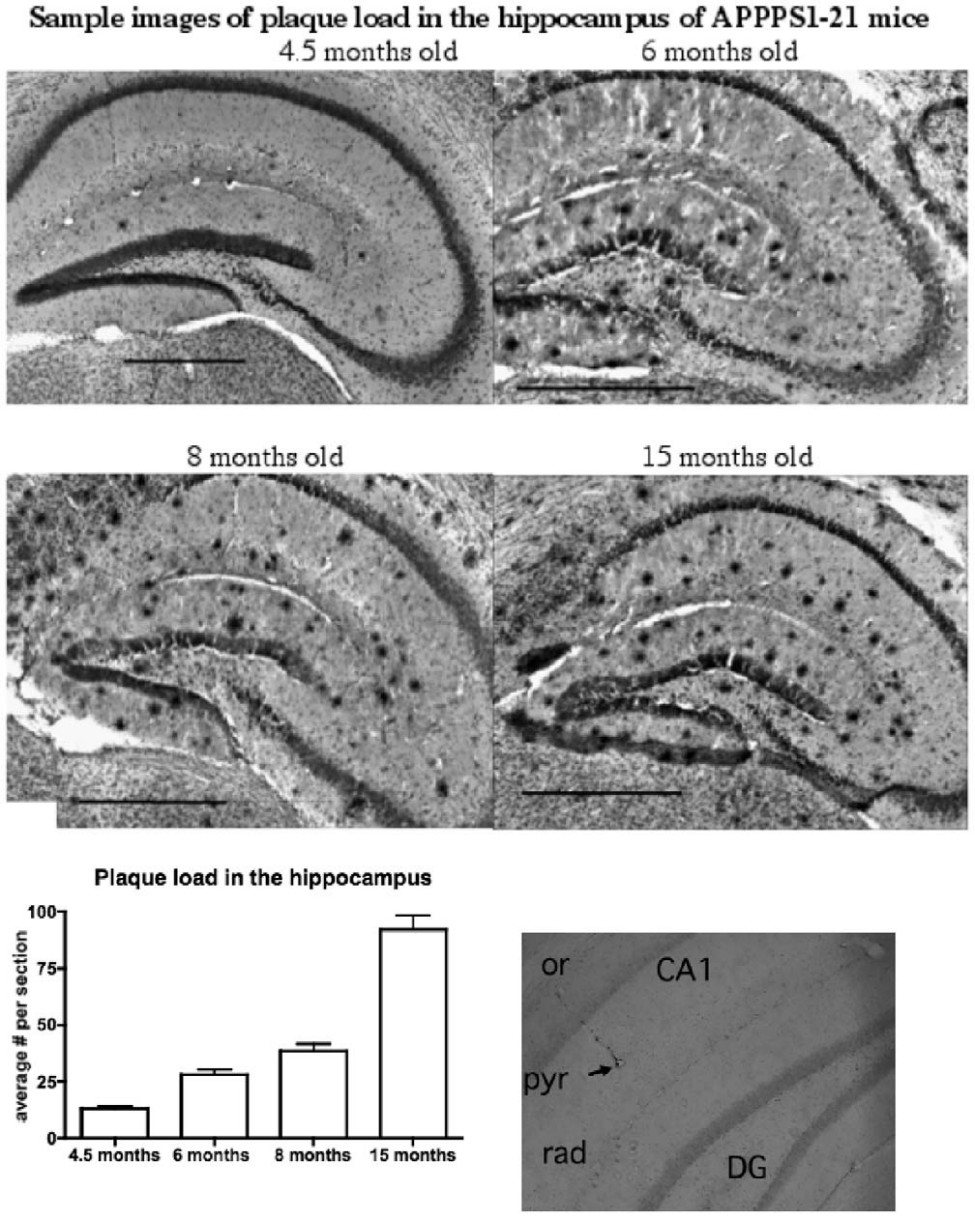
Analysis of the plaque load shows an age-dependent increase of plaque numbers in the hippocampus of APPPS1 mice. A one-way ANOVA showed an overall difference of p<0.0001. Scale bar  = 500 µm. Lower right: a sample micrograph of an electrode location in area CA1, strat. radiatum ( =  **rad**); **pyr**  =  strat. pyramidale, **or**  =  strat. oriens, **DG**  =  dentate gyrus.

Staining the beta-amyloid plaque load in the hippocampus showed a progressive increase of numbers in the APPPS1-21 mice. Controls showed no plaque staining. A one-way ANOVA revealed an overall difference between groups F = 104, p<0.0001 (see fig. 10).

## Discussion

The results presented here show that an expression of mutated human APP and PS-1 that produced β-amyloid1-42 and β-amyloid1-40 levels, plaque formation from 2–3 months onwards with the associated inflammatory response, and memory impairments from 8 months onwards [3] has distinct effects on synaptic plasticity in area CA1 of the hippocampus. In younger animals up to 5 months of age, no detrimental effects on synaptic plasticity were found. At this stage, β-amyloid production, plaque formation and the associated inflammation responses in the brain is still on a relatively low level. Significant amounts of plaques in the hippocampus were only observed from 6–8 months onwards, which is much delayed compared to the cortex. We previously observed that plaques in the cortex appear from 2 months onwards with a rapid increase in numbers, while the hippocampus showed a much delayed onset of amyloid deposition. In area CA1 of the hippocampus, increased plaque numbers did not appear until 8 months of age (see Radde et al. 2006 for examples of plaque deposition in the hippocampus and cortex), a finding that we confirmed in the present study. The relatively late impairment of CA1 LTP that we observed in the present study could be related to this delayed formation of plaques in the hippocampus. At 8 months of age, plaque formation had reached a comparatively high level, which could be responsible for the impairment in learning and LTP.

The central finding in this study is that LTP was completely obliterated in 8 and 15 month old APPPS1-21, while age matched wild type littermate controls still showed good LTP. This impairment of LTP can be due to several processes. At the 8 month time point, the learning ability of these mice is also severely compromised [3]. Other studies have shown that the APPPS1-21 mouse model has decreased numbers of synapses and also shows abnormal neuritic anatomy and regional neuronal loss when aged (Rupp et al., 2009), and a decrease in synapse numbers and vesicle release [20]. These processes will have detrimental effects on synaptic plasticity. Additionally, the inflammation response observed in these mice (Radde et al., 2009) will have further detrimental effects on synaptic plasticity through the release of proteolytic enzymes and the formation of reactive oxygen species [21].

While this strong impairment is to be expected, it does not explain why some mouse models that overexpress mutated APP and develop high numbers of plaques show no or very little impairment in synaptic plasticity [7], [9], [22]. There are several possible reasons for these differences. One LTP study that used the APP23 mice which also express the same Swedish APP mutation as the APPPS1-21 mice did not show any impairment in LTP. Somewhat surprisingly, the authors found a difference in basal transmission in the input-output analysis, but no differences in LTP even in aged 24 months old APP 23 mice (Roder et al., 2003). The study shows that a difference in baseline response does not necessarily translate into a difference in LTP. An important difference in the two mouse models is the amount of beta-amyloid and plaque load. The reason why the mutated PS1 had been added to the mutated APP gene was that plaque formation is very much accelerated by this. Plaques start to appear in the APPPS1-21 model at 2 months of age and numbers rapidly increase [3], while in the APP23 mouse model, plaques start to appear at 6 months of age [23]. The different genetic background also can have a large effect on physiological processes, since different mouse strains show different sensitivities to neurodegenerative influences (see [24] for a discussion of genetic background effects). In addition, there is evidence that not just the plaques themselves, but also soluble oligomers of β-amyloid have detrimental effects on synaptic transmission and neuronal survival. Different transgenic strains differ in the levels of production of amyoid oligomer species that have been shown to impair neuronal activity and synaptic plasticity [25]. Interestingly enough, there is also an increased risk for epilepsy in patients with dementia, making it likely that these compensatory processes are also found in human brains [26], [27]. These compensatory mechanisms, which are most likely controlled by a negative feedback system that keeps overall neuronal activity at physiological levels, could explain why some mouse models show remarkably few functional impairments and symptoms even with large plaque loads in the cortex. Consistent with these observations, imaging studies of Alzheimer patients and age matched controls showed no clear correlation between plaque load and cognitive impairment [28], indicating that there is a large variation between individuals in compensating for the plaque-associated gliosis, synaptic and neuronal loss, and other detrimental influences on brain activity. The mouse model analysed in this study not only has a mutated form of human APP, but also a mutated form of PS-1. PS-1 is involved in the cleavage of several cell signalling molecules, and an impairment of its function has effects on synaptic growth and vesicle release. In hippocampal cultures from APPPS1-21 mice, the size of the readily releasable synaptic vesicle pool was decreased, while in a single-mutant APP transgenic model (APP23, APPswe), this effect was not observed (Priller, 2009). Others have shown that in mice that express mutated PS-1, synaptic plasticity can be impaired [29]. However, in a different study that investigated PS-1 KO mice, little effect on LTP was found, while a APP(V717I) mutation mouse strain showed much impaired LTP. Deleting PS-1 in the APP(V717I) mice actually *rescued* LTP in these mice [30]. In a more recent study, mice that expressed mutant [A246E]PS-1 also showed healthy LTP which was even increased compared to controls [31]. These results suggest that the main effect observed in the LTP studies shown here are due to the overexpression and aggregation of mutated human APP, though a small contribution by the PS-1 mutation to this impairment cannot be ruled out.

At 6 months of age, somewhat surprisingly, a slight facilitation of PPF was observed in the APPPS1-21 animals. This finding is unexpected but may be explained as a form of compensation for synaptic impairment. Neuronal networks in the brain can compensate for impairments by upregulating excitatory or downregulating inhibitory neuronal activity. A study of APP-overexpressing mice strain (PDAPP mice) showed that in aged transgenic mice, baseline field potentials in the hippocampus often failed, while synaptic plasticity and LTP was often enhanced. The authors interpret the enhancement as a compensatory mechanism to counter-balance disturbances in synaptic transmission that appear when abundant plaques and Alzheimer's-like neuropathology are present, and which explains why such synaptic impairments are not necessarily accompanied by a disproportionate loss of LTP [22]. A different study has shown that GABAergic inhibition is altered in a APP/PS-1 mouse model of AD, showing that not only excitatory but also inhibitory activity is altered [32]. Therefore, it is possible that the increased PPF in the APPPS1-21 mice could be due to changed local GABA inhibition to compensate for a reduction in excitatory synaptic activity. The facilitatory effect of APP on PPF is lost at later stages of development in APPPS1-21 mice. PPF was not different in the 8 month old group compared with age matched littermate controls. At 15 months of age, a clear decrease of PPF was found in APPPS1-21 mice. The changes in PPF at the 160 ms interstimulus interval time point shown in our study also suggests that local inhibition of excitatory neuronal activity by GABAergic interneurons may be changed. It has been shown that PPF at the time window of around 160 ms is dependent on GABAb receptor activity, which are located on synapses of interneurons [13], [33]. However, further research using in vitro slices would need to confirm any changes in the GABAergic synaptic transmission in this mouse model, though one has to keep in mind that the spontaneous activity of neurons *in vitro* differs greatly from activity observed *in vivo*. If there is such a modulation of local inhibition, it could also explain why APP mouse models are reported to have an increased risk for epileptic seizures, which would be expected by a downregulation of inhibitory or an upregulation of excitatory neuronal activity [34].

In conclusion, the observed age- dependent impairment of synaptic plasticity in APPPS1-21 mice shows that the increasing levels of β-amyloid and accumulation of plaques with the associated gliosis and synaptic loss result in progressive deterioration of synaptic plasticity, which correlates with age-dependent memory impairments in this transgenic mouse strain. The APPPS1-21 mouse strain therefore appears to be a useful model for the study of APP and β-amyloid overexpression-related dysfunctions in synaptic activity and cognitive performance in the brain that is found in AD.
